# Fish Diversity and Functional Traits in the Seagrass Based on the Environmental DNA Metabarcoding in the Li’an Bay, China

**DOI:** 10.3390/ani16060871

**Published:** 2026-03-11

**Authors:** Weiwen Li, Weiyi He, Yanxu Zhang, Danyun Ou, Shangwei Wang, Yue Ni, Hao Huang, Ming Chen

**Affiliations:** Third Institute of Oceanography, Ministry of Natural Resources, Xiamen 361005, China; liweiwen@tio.org.cn (W.L.);

**Keywords:** fish diversity, environmental DNA, seagrass, multi-habitat

## Abstract

Environmental DNA metabarcoding has proven to be a powerful tool for monitoring fish diversity, and has been widely used in both freshwater and marine ecosystems. Fish diversity is a critical indicator to assess ecosystem function and its health. In this study, we took 12 samples from four areas (Area 1 (LS1) is the edge of the seagrass field adjacent to oyster reefs; Area 3 (LS3) is the edge of the seagrass field adjacent to coral reefs; Area 2 (LS2) is situated between LS1 and LS3 where there is dense seagrass distribution around the sampling sites; and Area 4 (LS4) is located between LS1 and LS3; we sample in the places at least 4 m by 4 m on the beach where no seaweed is present, each with three replicates) from Li’an Bay to detect fish diversity across multi-habitat seagrass areas based on the environmental DNA metabarcoding. A total of 312 Amplicon Sequence Variants (ASVs) were detected from 12 samples, and 58 fish species were annotated, representing 21 orders, 32 families and 48 generas. In general, the mean of Simpson and Pielou’s evenness indices followed the pattern LS1 > LS4 > LS2 > LS3, while the mean of Shannon–Wiener Diversity Index is LS4 > LS1 > LS2 > LS3. A total of 35 functional entities were observed among the four areas, with functional redundancy (FR) values of 0.800, 0.657, 0.542, and 0.7 for LS1, LS2, LS3, and LS4, respectively. All FR values were below 1.5, suggesting low ecological niche overlap among species within each area. These findings provide fundamental insights into the ecological health and stability of seagrass beds in Li’an Bay and offer a scientific basis for future ecological restoration efforts.

## Simple Summary

High fish diversity was observed in habitats with greater structural complexity areas. This study investigated fish diversity and functional traits across different habitat types within a seagrass bed. The results showed that the fish diversity was highest in multi-habitats—specifically where seagrass beds were adjacent to oyster reefs, while both fish diversity and functional richness were lowest in areas lacking seagrass. The seagrass beds in the Li’an Bay region have been negatively impacted by human activities. Our findings provide a scientific basis for future ecological restoration efforts in the seagrass ecosystems of Li’an Bay.

## 1. Introduction

Fish diversity is a critical indicator for assessing the health of marine ecosystems. However, fish diversity is experiencing a rapid decline due to anthropogenic activities [1] or climate change [2]. Traditional methods for fish diversity assessment, such as trawling and gillnetting, are time-consuming, invasive, and often fail to detect rare or elusive species [3]. Moreover, sampling difficulties significantly reduce the probability of identifying these species, making quantitative monitoring of fish assemblages both challenging and costly. Therefore, there is a growing need for efficient and non-invasive survey methods. eDNA refers to genetic material obtained directly from environmental samples, such as water, without the need to capture organisms. It originates from sources including metabolic waste, skin cells, and excrement [4]. Despite ongoing debates about its ability to indicate species abundance or the presence of living organisms [5], eDNA metabarcoding has been widely recognized as an effective, non-invasive tool for monitoring biodiversity [6], including fish diversity. It has been applied successfully in both freshwater [7,8] and marine environments [9,10], and is especially promising for use in marine protected areas [11].

Li’an Bay, China, hosts the nation’s first seagrass marine protected area, established in 2015 under the jurisdiction of the Hainan Ocean and Fisheries Department. The area is known for its wide distribution of seagrass beds and rich biodiversity. However, these ecosystems face increasing pressure from overexploitation, land-based pollution, unsustainable fishing and tourism, and climate-induced sea-level rise. These factors threaten the biological diversity and structural stability of the seagrass ecosystem. Due to the cryptic nature of many fish species and the limitations of traditional survey techniques, few studies have evaluated fish diversity and functional diversity in Li’an Bay. One exception is an acoustic survey conducted from 2014 to 2016 in Lingshui Bay, which recorded 65 to 81 fish species [12]. Recent studies have demonstrated that eDNA metabarcoding is effective for detecting cryptic, rare, or transient species, offering a more complete view of fish communities and functional traits in marine protected areas [13,14,15].

**Figure 1:**
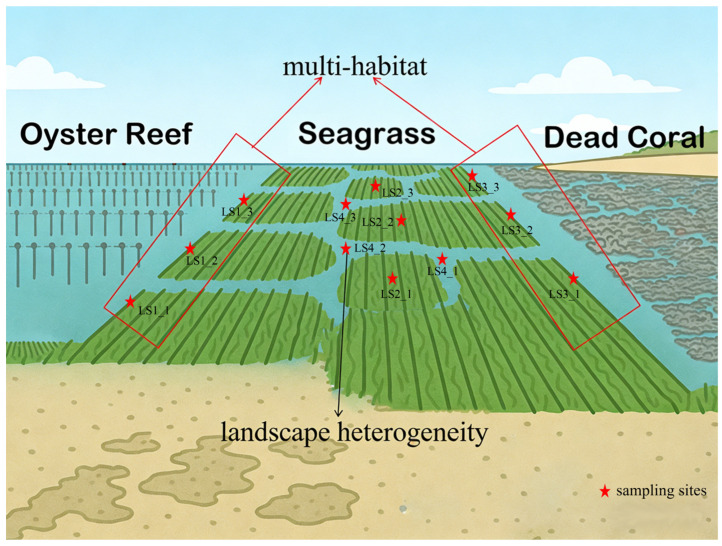
The sketch map of multi-habitat and landscape heterogeneity.

**Figure 2:**
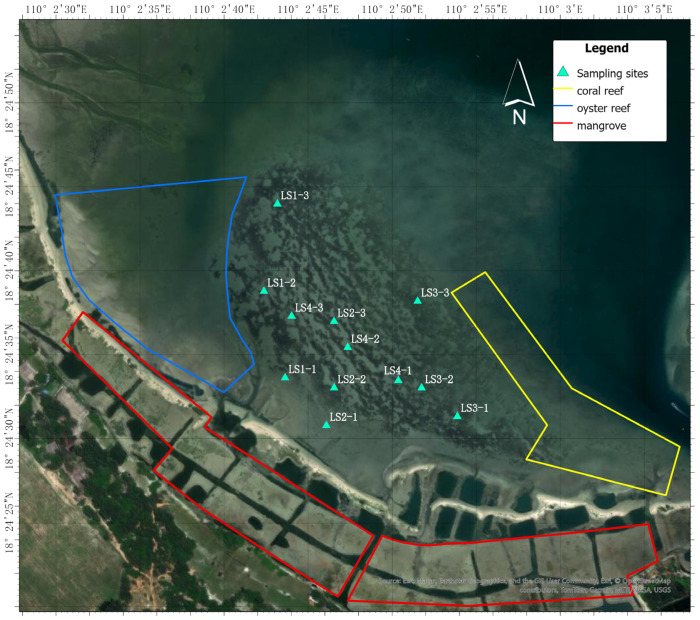
Sampling locations in the Li’an Bay.

In this study, eDNA samples were collected in April 2024 from the seagrass marine protected area in Li’an Bay. Environmental variables such as temperature, salinity, dissolved oxygen, total nitrogen, total phosphorus, and total organic carbon were measured simultaneously. The objectives of this study were to: (1) evaluate the hypothesis that fish and functional diversity are higher in multi-habitats (Figure 1), and (2) assess whether landscape heterogeneity (Figure 1) contributes to increased fish and functional diversity.

## 2. Material and Methods

### 2.1. Sampling Site

Li’an Bay is a natural semi-enclosed lagoon located in Hainan Province, China. The lagoon spans from 110.04° E to 110.06° E and from 18.40° N to 18.44° N, covering an area of approximately 9.00 km^2^, with water depths ranging from 1.90 to 7.60 m. The sampling site is bordered by a shipping area to the north, mangroves to the south, oyster reefs to the west, and coral reefs to the east. Four sampling areas were designated: Area 1 (LS1) is the edge of the seagrass adjacent to oyster reefs; Area 3 (LS3) is the edge of the seagrass adjacent to coral reefs; Area 2 (LS2) is situated between LS1 and LS3 where there is dense seagrass distribution around the sampling sites; and Area 4 (LS4) is located between LS1 and LS3; we sample in the places at least 4 m by 4 m on the beach where no seaweed is present (Figure 1 and Figure 2).

### 2.2. eDNA Sampling

The eDNA sampling campaign was conducted in April 2024. Four areas were sampled, each consisting of three sites: LS1 (LS1_1, LS1_2, LS1_3), LS2 (LS2_1, LS2_2, LS2_3), LS3 (LS3_1, LS3_2, LS3_3), and LS4 (LS4_1, LS4_2, LS4_3). All water samples were collected from a depth of approximately 1 m.

### 2.3. Sample Processing

At each site, 500 mL of surface seawater was collected using a water sampler and stored in sterile sampling bags (BKMAN, Qingdao, China), then transported in a 4 °C insulated container. Water was filtered through a 0.2 μm pore size polycarbonate membrane (Millipore, Billerica, MA, USA), and filters were transferred to 1 mL cryogenic storage tubes (Axygen, Union City, CA, USA), followed by immediate preservation in liquid nitrogen. Environmental parameters, including dissolved oxygen (DO), chlorophyll-a (Chla), salinity, and turbidity, were measured in situ using a ProDSS Water Quality Monitoring System (YSI, Yellow Springs, OH, USA). All equipment was rinsed with Milli-Q water before use.

### 2.4. DNA Extraction Process

DNA was extracted using the DNeasy PowerWater Kit (QIAGEN, Hilden, Germany) following the manufacturer’s protocol.

### 2.5. Amplicon Library and Sequencing

PCR amplification targeted the 12S rRNA gene using primers MiFish-U-F (5′-GTYGGTAAAWCTCGTGCCAGC-3′) and MiFish-U-R (5′-CATAGTGGGGTATCTAATCCYAGTTTG-3′), with sample-specific 7-bp barcodes. The 25 μL PCR reaction contained: 5 μL Q5 Reaction Buffer (5×), 5 μL Q5 High-Fidelity GC Buffer (5×), 0.25 μL Q5 DNA Polymerase (5 U/μL), 2 μL dNTPs (2.5 mM), 1 μL of each primer (10 μM), 2 μL template DNA, and 8.75 μL nuclease-free water. Thermal cycling conditions included an initial denaturation at 94 °C for 2 min, followed by 35 cycles at 98 °C for 5 s, 50 °C for 10 s, and 72 °C for 10 s, with a final extension at 72 °C for 5 min [16]. PCR products were purified using Agencourt AMPure XP Beads (Beckman Coulter, Brea, CA, USA), quantified with the PicoGreen dsDNA Assay Kit (Invitrogen, Carlsbad, CA, USA), pooled in equimolar concentrations, and sequenced on the Illumina NovaSeq platform (2 × 300 bp) using the NextSeq™ 2000 SP Reagent Kit (500 cycles) by Shanghai Personal Biotechnology Co., Ltd. (Shanghai, China).

### 2.6. Quality Control and Assembling of MiSeq Reads

Bioinformatics analysis was conducted using QIIME2 (version 2022.11) [17]. Raw sequences were demultiplexed with the demux plugin, and primers were trimmed using cutadapt [18]. Sequence quality filtering, denoising, paired-end read merging, and chimera removal were performed using the DADA2 plugin. Singletons were removed before downstream analyses.

### 2.7. Taxonomic Assignment and ASVs

Unique sequences were clustered at 98% similarity using the cluster_size algorithm, and chimeric sequences were removed using uchime_denovo. Remaining sequences were reclustered at 100% similarity to generate Amplicon Sequence Variants (ASVs). Non-singleton ASVs were aligned with MAFFT [19], and a phylogenetic tree was constructed using FastTree2 (version v2.1.11) [20]. Taxonomic assignment was performed using the classify-sklearn classifier in QIIME2’s feature-classifier plugin [21], referencing the NCBI database (https://www.ncbi.nlm.nih.gov). During taxonomic assignment, multiple metrics were considered to select the most appropriate match, including alignment length, percent identity, the number of mismatches within the aligned region, bit score, and E-value. These metrics were combined to derive an overall score for identifying the best hit, and assignments preferentially supported sequences showing 100% identity, a higher bit score, and the lowest E-value [22]. Hits with E-values greater than 1 × 10^−5^ were designated as unassigned [22]. When an ASV matched multiple highly similar sequences, the final taxon was chosen using the combined evaluation of bit score and E-value, with additional refinement based on ecological plausibility using species distribution information from FishBase (https://fishbase.se/search.php, accessed on 27 May 2024). All taxa identified were subsequently checked against FishBase to verify their distribution ranges. To improve reliability, species not documented in FishBase for the study area and lacking confirmation were removed from the species list. Unassigned ASVs were excluded [23].

### 2.8. Fish Diversity and Functional Diversity Analysis

All analyses were conducted using R software (version 4.3.3) [24]. Ecological community analysis was performed with the vegan package (version 2.7.2) [25]. Sequencing depth across samples was approximately 60,000 reads per sample. Sequencing depth was assessed in QIIME2 by performing random subsampling of the ASV abundance table across a range of sequencing depths to generate rarefaction curves, which was used to evaluate whether the sequencing effort was sufficient to capture sample diversity. To account for variation in sequencing depth among samples at the ASV level for downstream diversity comparisons, the ASV abundance table was then rarefied in QIIME2 by random subsampling without replacement to a common depth defined as 95% of the read count of the sample with the lowest sequencing depth. Species abundance was determined based on the number of sequencing reads obtained from eDNA metabarcoding. To evaluate alpha diversity and evenness, several ecological indices were calculated. The Shannon diversity index was computed using the diversity function, and Pielou’s evenness was derived by dividing the Shannon index by the natural logarithm of species richness. The Simpson index was calculated using the same function with index = “simpson”. Species richness (observed species) was calculated by counting the number of ASVs with non-zero abundance using rowSums (ASV > 0).

Hierarchical clustering of sampling stations was performed for the heat map of fish species composition in Lian Bay based on the Bray–Curtis dissimilarity matrix with the method of average.

The functional structure of fish communities was assessed to characterize the ecological roles and trait distribution of species [26]. Functional diversity (FD), which reflects species’ trait variation and their interactions with the environment, was assessed using trait data retrieved from FishBase (https://www.fishbase.org), including habitat type, water column position, trophic level, migratory behavior, and maximum body length [27]. The mFD R package (version 1.0.7) was used to calculate functional diversity indices based on species abundance and trait matrices [28]. The indices included Functional Richness (FRic), Functional Dispersion (FDis), Functional Divergence (FDiv), Functional Evenness (FEve), Functional Originality (FOri), Functional Specialization (FSpe), Functional Mean Pairwise Distance (FMPD), and Functional Nearest Neighbor Distance (FNND). FRic quantifies the functional space occupied by the community as the volume of the convex hull enclosing species, computed only when species number exceeds trait dimensions. FDis measures the weighted deviation of species from the community’s centroid. FDiv assesses the distribution of species’ biomass relative to the convex hull vertices. FEve evaluates the evenness of biomass distribution along the minimum spanning tree among species. FOri calculates the mean distance from each species to its nearest species in the global species pool. FSpe quantifies the mean distance from each species to the centroid of the global species pool. FMPD reflects the average distance among all species pairs in the community. FNND measures the distance to the nearest neighbor within the community. Functional Entities (FEs), defined as unique combinations of functional traits, were identified using the species_to_FE function. Functional Redundancy (FR) was calculated as the ratio of species richness (S) to the number of functional entities (FEs), i.e., FR = S/FE [29]. This metric reflects the extent to which multiple species share similar ecological functions.

## 3. Results

### 3.1. MiSeq Sequencing and Assignment

All samples were successfully amplified using MiFish primers. A total of 1,016,691 raw reads were obtained. After quality filtering and processing, 920,029 non-singleton reads were retained for analysis, with an average of 76,669 ± 6615 reads per sample (Table 1). Following standard quality control and sequence processing, 312 amplicon sequence variants (ASVs) were identified across 12 samples. Among them, 58 fish species were taxonomically annotated, belonging to 21 orders, 32 families, and 48 genera (Table 2). The most abundant genera detected by the MiFish primers included *Favonigobius*, *Fibramia*, *Terapon*, *Cryptocentrus*, *Pelates*, *Pristigenys*, *Callionymus*, *Drombus*, *Pristiapogon*, and *Paracentropogon*.

### 3.2. Spatial Variation in Fish Communities

**Figure 3:**
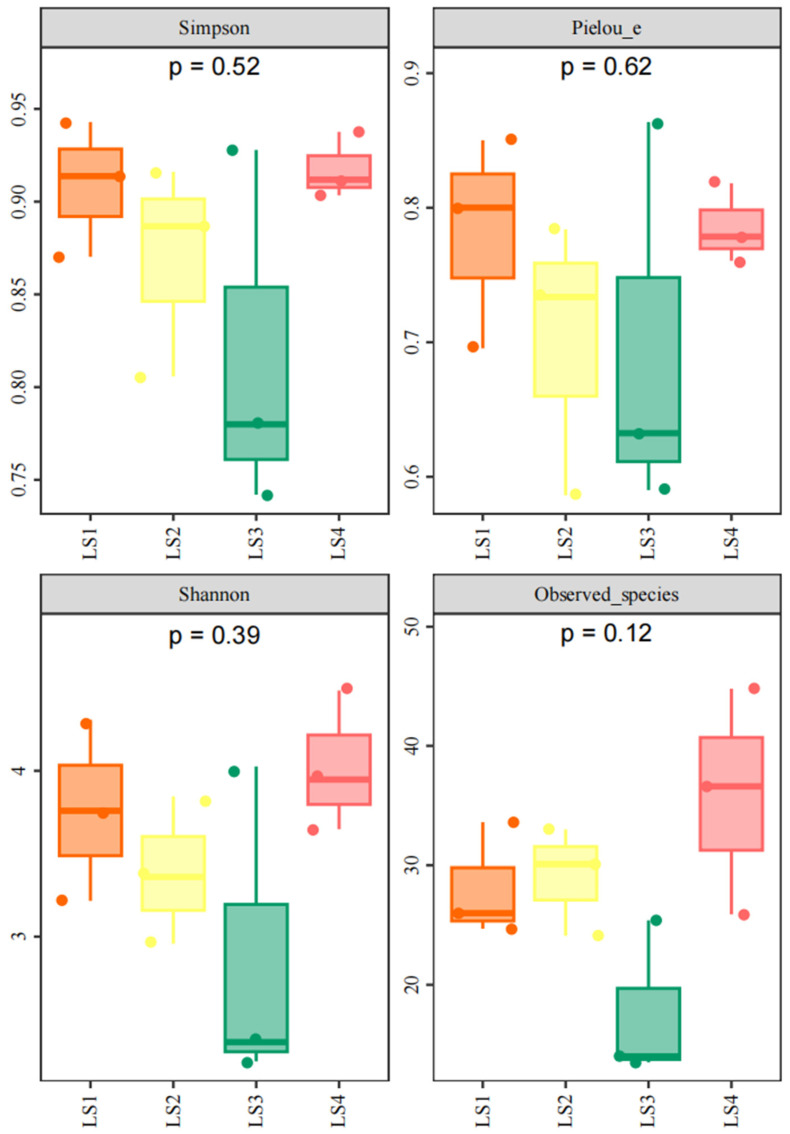
α-diversity parameters among 4 sampling areas.

The relative abundance of fish species varied across the four sampling lines. Although alpha diversity indices—including Chao1, observed species richness, Shannon index, and Simpson index—did not show statistically significant differences among the sampling lines (*p* > 0.05, Figure 3), according to the collected data, the means of Shannon index among 4 sampling areas are LS1 > LS2 > LS3, with LS4 showing values similar to LS1.

**Figure 4:**
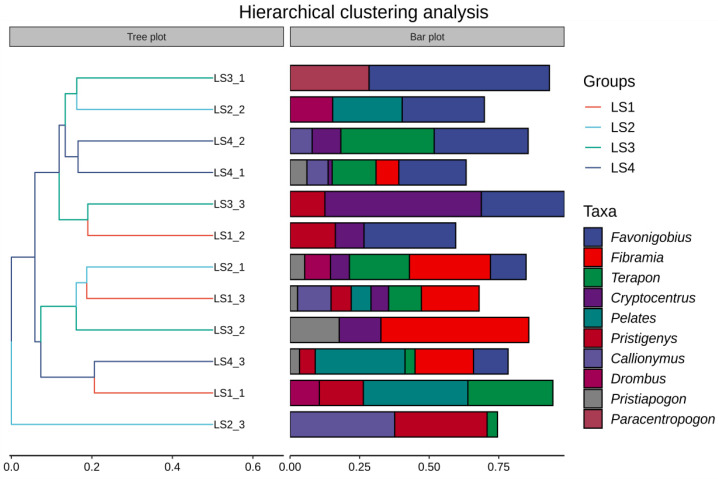
Heat map of fish species composition in the Li’an Bay.

The hierarchical clustering (Figure 4) revealed spatial variation in fish community composition. The clustering results show that the results of all sites cluster into 3 branches. Sampling sites LS2_1, LS1_3, LS3_2, LS4_3 and LS1_1 clustered together, while LS3_1, LS2_2, LS4_2, LS4_1, LS3_3 and LS1_2 clustered together. LS2_3 was apart from the above two branches, and becomes an independent branch. The irregular clustering suggests variation in species composition.

### 3.3. Functional Traits and Their Spatial Patterns

**Figure 5:**
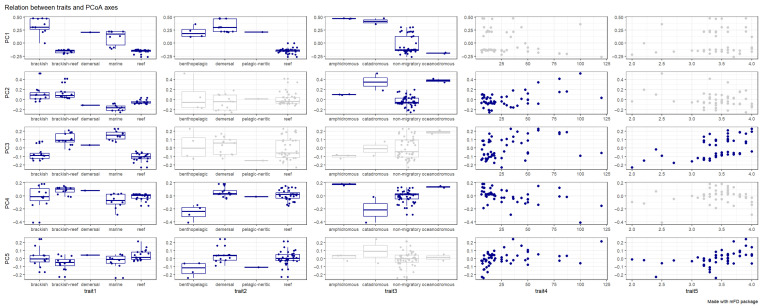
The relation between traits and PCoA axes. Note: habitat preference, vertical distribution in the water column, migratory behavior, maximum body length and trophic level were used to analysis.

PCoA revealed distinct associations between functional traits and community structure. The first principal coordinate (PC1) exhibited strong correlations with habitat preference (trait 1: Kruskal–Wallis η^2^ = 0.742, *p* < 0.01), vertical distribution in the water column (trait 2: η^2^ = 0.660, *p* < 0.01), and migratory behavior (trait 3: η^2^ = 0.279, *p* < 0.01; Figure 5). In contrast, PC2 was primarily associated with habitat preference (trait 1: η^2^ = 0.789, *p* < 0.01) and migratory behavior (trait 3: η^2^ = 0.252, *p* < 0.01), while also showing significant relationships with maximum body length (trait 4: linear regression *R*^2^ = 0.299, *p* < 0.01) and trophic level (trait 5: *R*^2^ = 0.090, *p* < 0.05; Figure 5).

**Figure 6:**
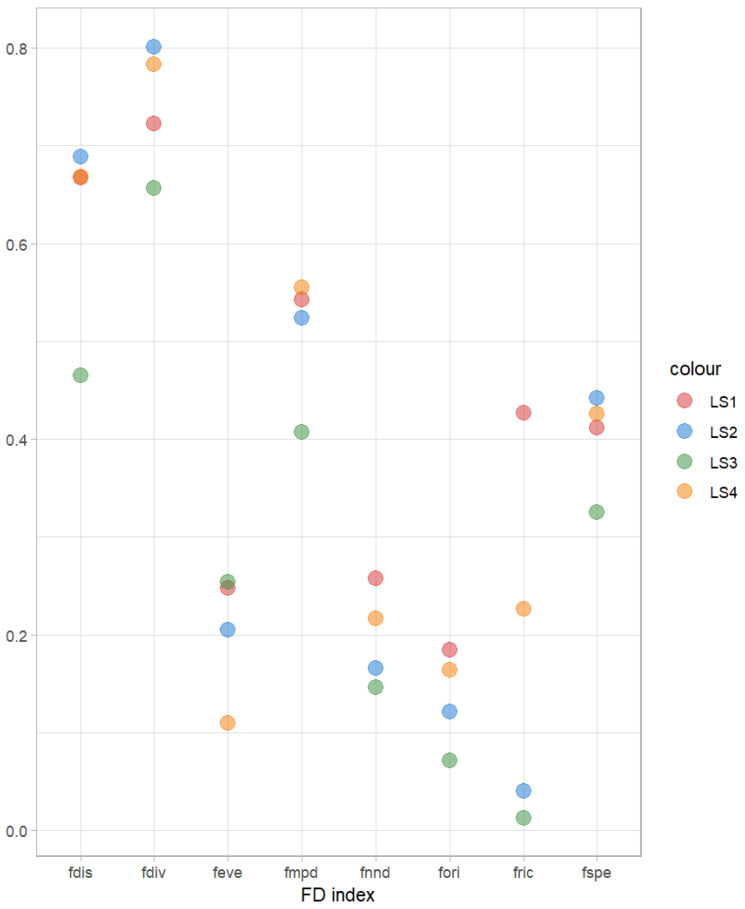
Functional traits among 4 sampling areas. Note: Functional traits including Higher Functional Divergence (fdiv), Functional Mean Pairwise Distance (fmpd), Functional Nearest Neighbor Distance (fnnd), Functional Richness (fric) and Functional Originality (fori).

Higher Functional Divergence (fdiv) values observed at LS1 and LS2 suggest that these locations harbor species with more extreme functional traits, and a strong ecological niche complementarity of species in the community, but the competition is low (Figure 6). In contrast, the low Functional Mean Pairwise Distance (fmpd) value at LS3 indicates limited functional differentiation among species, implying functional similarity in the community (Figure 6). Similarly, the Functional Nearest Neighbor Distance (fnnd) values at LS2 and LS3 were lower than those at other sampling lines, reflecting a more clustered distribution of species in the functional space (Figure 6). The Functional Richness (fric) value at LS1 was higher than those of the other sampling lines, indicating a greater occupancy of functional trait space and a higher level of functional diversity at this location (Figure 6). Conversely, the Functional Originality (fori) value at LS3 was the lowest among all sites, suggesting that the species present in this community are functionally less unique, with more common or redundant trait combinations (Figure 6).

### 3.4. Functional Redundancy of Fish Communities

**Figure 7:**
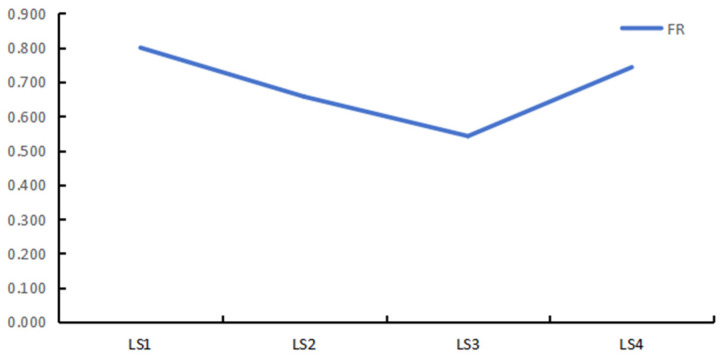
Functional redundancy among 4 sampling areas.

Functional redundancy analysis identified 35 FEs across the four sampling locations. The calculated FR values for LS1, LS2, LS3, and LS4 were 0.800, 0.657, 0.542, and 0.743, respectively—all below the threshold of 1.5, indicating a low level of functional redundancy within these fish communities. This suggests that the ability of fish species to compensate for one another in maintaining ecosystem functions is limited. Among the four locations, the capacity for functional compensation followed the pattern: LS1 > LS4 > LS2 > LS3 (Figure 7), indicating that LS1 exhibited the highest potential for ecological function persistence in the face of species loss, whereas LS3 showed the lowest.

## 4. Discussion

Fish play a crucial role in maintaining marine ecosystem balance and are often used as indicator species. In recent years, environmental DNA (eDNA) metabarcoding has emerged as a non-invasive and efficient method for assessing fish diversity in various marine environments, including estuaries [30], coastal zones [31], bays [32], and deep-sea ecosystems [33]. In this study, we applied eDNA metabarcoding to investigate fish diversity across multi-habitat areas within the seagrass beds of Li’an Bay, China. The presence of fish in seagrass beds plays a vital role in supporting ecosystem functions, making them important indicators of habitat health. Our results offer baseline data for evaluating the ecological condition of protected seagrass habitats and assessing human impacts.

Li’an Bay is a designated seagrass protection area, but its marine ecosystem has been significantly affected by anthropogenic activities, leading to declines in macrobenthic abundance and biomass [34]. Acoustic surveys conducted between 2014 and 2016 in the Lingshui Bay region recorded 65 to 81 fish species [12]. In our study, 58 fish species belonging to 21 orders, 32 families, and 48 genera were identified, 23 fish species fewer than that recorded by acoustic surveys. However, this discrepancy also may be due to limitations in the eDNA reference database [35]. To reduce potential methodological bias, rarefaction of the ASV table to a common sequencing depth prior to diversity calculations minimizes bias from unequal sequencing effort, while singleton removal reduces the influence of extremely rare ASVs that likely reflect stochastic noise, thereby improving the robustness of abundance-sensitive diversity metrics. Nevertheless, eDNA metabarcoding provided valuable new insights into fish diversity and offers a promising tool for biodiversity assessment. These results would provide datasets to evaluate fish diversity affected by the human activities.

Fish diversity detected via eDNA metabarcoding is strongly correlated with both species richness and abundance [36,37]. Habitat complexity has been widely shown to enhance fish diversity [38,39]. In our study, fish diversity followed the pattern LS1 > LS3 > LS2, with LS4 showing similar diversity levels to LS1. LS1, located at the interface between oyster reefs and seagrass beds, exhibited the highest fish diversity, likely due to habitat overlap [40]. LS4, also associated with a complex landscape, had slightly lower diversity than LS1. In contrast, LS3—near a degraded coral reef—exhibited reduced diversity, and LS2, located in a homogenous seagrass zone, showed the lowest diversity due to limited habitat heterogeneity.

Functional diversity provides a measure of the redundancy and complementarity within fish communities and can reflect ecosystem responses to environmental changes and anthropogenic pressures [41,42]. High functional diversity is often associated with high habitat heterogeneity [43,44]. Our findings show that the mean of functional diversity also followed the pattern LS1 > LS4 > LS3 > LS2, consistent with the pattern of taxonomic diversity. Both Callionymus and Pristigenus species prefer habitats with shelter and relatively clear water [45,46]. Seagrass provides shelter for these species. LS2_3 is located at the seagrass edge which is relatively far from the beach area, and the water is relatively clearer. This unique marine environment results in higher abundance of Callionymus and Pristigenus at LS2_3. Although this trend is not statistically significant, the mean of three parallel sampling results demonstrates a consistent pattern of this change, aligning with previous research findings [47]. The absence of seasonal statistical data may have contributed to this non-significant difference. Therefore, we believe the results of this study partially support the correlation between habitat complexity and fish functional diversity, which might suggest that multi-habitat areas support both higher species and functional trait diversity.

Li’an Bay’s protected seagrass beds face pressures from tourism and fishing, particularly during low tides. According to tourism statistics from Hainan Province’s Department of Tourism, Culture, Radio, Television and Sports, overnight tourist numbers in March to May 2024 showed year-on-year growth of 8.3%, −2.5%, and 17.2% respectively (https://lwt.hainan.gov.cn/xxgk_55333/lytj/2024lytj/, accessed on 17 September 2024). With the increase in tourist numbers, there is more and more tourism activity among the Li’an Bay’s protected seagrass beds. Previous studies have shown that fish diversity and functional traits are sensitive to human interference [48,49] and climate change [50,51]. Our functional redundancy analysis identified 35 functional entities across sampling sites. The functional redundancy (FR) values for LS1, LS2, LS3, and LS4 were 0.8, 0.657, 0.542, and 0.743, respectively—all below 1.5—indicating low redundancy [52,53]. This suggests that the loss of functionally similar species could significantly impair ecosystem resilience. Fish functional traits were impacted by human activities and climate change [48,49,50,51], with climate change having a gradual and long-term effect on functional traits, while human interference is relatively intense and immediate. During our field sampling collection, many locals and tourists are actively among the protected sea areas. Therefore, we conclude that human activities may contribute to reduced functional diversity, but further quantitative evidence is needed.

Seagrass habitats are known hotspots of fish biodiversity [54] and functional trait diversity [55]. When relatively undisturbed, seagrass meadows can recover quickly through active restoration and reduced human activity. eDNA metabarcoding has been proved as a useful environmental monitoring tool for assessing ecological health and stability [56,57]. This study did not consider seasonal and annual variations, which may overlook the seasonal variation of fish communicates. Given the observed low fish and functional diversity in parts of Li’an Bay and the significant human activity pressure faced by the protected areas, we recommend strengthening conservation efforts, including restricting human access to sensitive areas and implementing landscape-scale seagrass restoration. Such actions are essential for enhancing both species diversity and functional resilience in this protected marine ecosystem

## 5. Conclusions

In this study, a total of 58 fish species were detected, belonging to 21 orders, 32 families, and 48 genera. Alpha diversity indices, including Chao1, Observed Species, Shannon Index, and Simpson Index, showed no significant differences among the sampling lines. Overall, the Simpson Index and Pielou’s Evenness Index followed the pattern LS1 > LS4 > LS2 > LS3, while the Shannon–Wiener Index showed the pattern LS4 > LS1 > LS2 > LS3. Hierarchical clustering analysis indicated that the distribution of fish in the seagrass meadow was relatively uniform across sampling locations. In total, 35 functional entities were identified, with FR values ranging from 0.542 to 0.800, suggesting low redundancy among fish communities in the study area.

## Figures and Tables

**Table 1:** Sequences’ data process distribution among each sites.

Sample ID	Input	Filtered	Denoised	Merged	Non-Chimeric	Non-Singleton
LS1_1	79,093	75,614	74,721	74,454	72,354	72,250
LS1_2	86,254	83,756	82,762	82,343	79,871	79,717
LS1_3	81,920	79,108	78,150	77,600	75,370	75,227
LS2_1	78,542	75,514	73,601	71,136	67,674	67,170
LS2_2	97,479	94,405	93,682	92,782	90,635	90,532
LS2_3	84,953	79,649	78,742	76,159	73,972	73,667
LS3_1	90,553	85,121	83,800	83,188	80,859	80,651
LS3_2	73,147	70,609	69,959	69,445	67,739	67,661
LS3_3	85,779	83,033	81,852	81,314	78,819	78,651
LS4_1	88,578	85,690	84,495	83,625	80,524	80,321
LS4_2	90,341	87,952	86,264	85,152	82,152	81,803
LS4_3	80,052	77,700	76,335	75,298	72,664	72,379
Total	1,016,691	978,151	964,363	952,496	922,633	920,029

**Table 2:** Detection of fish species in Li’an Bay based on eDNA metabarcoding technology.

Taxon	LS1	LS2	LS3	LS4
*Acanthurus nigros*	+	−	−	−
*Uroconger lepturus*	+	+	−	−
*Moringua javanica*	−	+	−	−
*Gymnothorax buroensis*	−	+	−	−
*Petroscirtes breviceps*	+	−	−	+
*Petroscirtes mitratus*	+	−	−	+
*Pelates quadrilineatus*	+	+	−	+
*Terapon jarbua*	+	+	−	+
*Chaetodon punctofasciatus*	+	−	−	−
*Coradion altivelis*	−	−	+	−
*Heniochus chrysostomus*	+	−	−	+
*Heniochus varius*	+	−	−	−
*Prognathodes aya*	+	+	+	+
*Sardinella hualiensis*	−	−	−	+
*Thryssa encrasicholoides*	−	+	−	−
*Gerres shima*	+	−	−	+
*Acentrogobius multifasciatus*	−	−	−	+
*Asterropteryx semipunctata*	−	−	+	−
*Cryptocentroides insignis*	−	+	−	−
*Cryptocentrus caeruleomaculatus*	−	−	−	+
*Cryptocentrus melanopus*	+	+	+	−
*Cryptocentrus nigrocellatus*	−	−	+	−
*Drombus triangularis*	+	+	−	−
*Favonigobius melanobranchus*	+	+	+	+
*Favonigobius reichei*	+	−	−	−
*Myersina filifer*	−	−	+	−
*Fibramia amboinensis*	+	+	+	+
*Ostorhinchus aureus*	−	+	+	−
*Pristiapogon exostigma*	+	+	+	+
*Cheilio inermis*	−	−	−	+
*Halichoeres podostigma*	−	−	+	−
*Hapalogenys kishinouyei*	+	−	+	+
*Plectorhinchus sordidus*	−	−	−	+
*Moolgarda engeli*	+	−	−	+
*Mugil cephalus*	+	−	−	+
*Carapus boraborensis*	+	−	−	+
*Platycephalus indicus*	+	+	+	+
*Epinephelus quoyans*	−	−	−	+
*Liopropoma aragai*	−	+	−	−
*Paracentropogon longispinis*	-	+	−	−
*Paracentropogon rubripinnis*	-	+	−	−
*Crossorhombus kanekonis*	+	−	−	−
*Cynoglossus quadrilineatus*	+	+	+	+
*Paraplagusia japonica*	+	−	−	−
*Pardachirus pavoninus*	-	−	+	+
*Pristigenys niphonia*	+	+	+	+
*Lethrinus microdon*	+	−	−	−
*Lethrinus nebulosus*	+	−	−	−
*Rhabdosargus sarba*	-	−	−	+
*Callionymus enneactis*	-	+	+	+
*Callionymus simplicicornis*	+	+	−	−
*Upeneus japonicus*	-	+	+	−
*Hippichthys cyanospilos*	-	+	+	−
*Odonus niger*	-	+	+	+
*Arothron hispidus*	+	−	−	+
*Arothron meleagris*	+	−	−	−
*Arothron stellatus*	+	−	−	−
*Cyttopsis cypho*	+	+	−	−

Notes: + indicates that the fish has been detected in the sampling line, while − indicates that the fish were not detected.

## Data Availability

The data presented in this study are available upon request from the corresponding author.
